# Effects of plasma viscosity modulation on cardiac function during moderate hemodilution

**DOI:** 10.4103/0973-6247.67034

**Published:** 2010-07

**Authors:** Surapong Chatpun, Pedro Cabrales

**Affiliations:** 1 *Department of Bioengineering, University of California, San Diego, La Jolla, CA, USA*; 2 *Institute of Biomedical Engineering, Prince of Songkla University, Songkhla, Thailand*

**Keywords:** Cardiac function, hemodilution, plasma expander, plasma viscosity

## Abstract

**Background:**

Previous studies have found that increasing plasma viscosity as whole blood viscosity decrease has beneficial effects in microvascular hemodynamics. As the heart couples with systemic vascular network, changes in plasma and blood viscosity during hemodilution determine vascular pressure drop and flow rate, which influence cardiac function. This study aimed to investigate how changes in plasma viscosity affect on cardiac function during acute isovolemic hemodilution.

**Materials and Methods:**

Plasma viscosity was modulated by hemodilution of 40% of blood volume with three different plasma expanders (PEs). Dextran 2000 kDa (Dx2M, 6.3 cP) and dextran 70 kDa (Dx70, 3.0 cP) were used as high and moderate viscogenic PEs, respectively. Polyethylene glycol conjugated with human serum albumin (PEG-HSA, 2.2 cP) was used as low viscogenic PE. The cardiac function was assessed using a miniaturized pressure-volume conductance catheter.

**Results:**

After hemodilution, pressure dropped to 84%, 79%, and 78% of baseline for Dx2M, Dx70 and PEG-HSA, respectively. Cardiac output markedly increased for Dx2M and PEG-HSA. Dx2M significantly produced higher stroke work relative to baseline and compared to Dx70.

**Conclusion:**

Acute hemodilution with PEG-HSA without increasing plasma viscosity provided beneficial effects on cardiac function compared to Dx70, and similar to those measured with Dx2M. Potentially negative effects of increasing peripheral vascular resistance due to the increase in plasma viscosity were prevented.

## Introduction

Blood losses are usually corrected initially by the restitution of volume with plasma expanders (PEs), and subsequently by the restoration of oxygen carrying capacity by means of either a blood transfusion. This procedure has been validated in many studies and is embodied in the practice of hemodilution.[1–3] The presumed universal benefit of instituting hemodilution with low viscosity PEs was challenged by Tsai *et al*,[4] who showed that microvascular function can only be maintained in extreme hemodilution if plasma viscosity is increased during the hemodilution process. This approach showed that in the awake hamster window chamber model hemodilution could be carried to 11% hematocrit (Hct), while preserving normal capillary flow if plasma viscosity was elevated to about 2.2 cP using dextran 500 kDa (Dx500). Conversely, capillary flow could not be maintained at a systemic hematocrit of 11% if plasma viscosity was about 1.1 cP as attained when dextran 70 kDa (Dx70) was used as the hemodilutent.

Recently, it has been proposed that the conjugation of protein with polyethylene glycol (PEG) such as PEG-conjugated human serum albumin (PEG-HSA) and hemoglobin (PEG-Hb) may be the next step of PEs.[5–8] The conjugation of protein with PEG provides longer circulating time, lowers the toxicity and immunologic reactions, and increases solubility in water. Cabrales *et al*,[8] performed an extreme hemodilution with PEG-Hb and PEG-HSA in an awake hamster window model and found that these PEG compounds maintained microvascular conditions with lower concentrations than conventional PEs such as Dx70. These results were similar when using high viscogenic PEs, alginate and Dx500, compared with Dx70 in an extreme hemodilution model.[9]

The aim of this study was to evaluate the effect of change in plasma viscosity on cardiac function with PEG-HSA (viscosity; 2.2 cP) compared to 6% dextran 2000 kDa (viscosity; 6.3 cP) and 6% dextran 70 kDa (viscosity; 3.0 cP) by using an acute moderate isovolemic hemodilution (Hct 28%) in anesthetized hamsters. Moderate isovolemic hemodilution was performed by a 40% blood volume exchange with test PEs followed by 1 hr monitoring period. To accomplish this goal, we evaluated left ventricular function indices derived from the pressure-volume (PV) measurement using a miniaturized PV conductance catheter. This technique provides a real-time ventricular volume measurement and a benefit to simultaneously quantify load and interaction between heart and vasculature in each cardiac cycle.

## Materials and Methods

### Animal preparation

Studies were performed in anesthetized male Golden Syrian hamsters (Charles River Laboratories; Boston, MA) weighing 60-70 g. Animal handling and care followed the NIH Guide for Care and Use of Laboratory Animals. The experimental protocol was approved by the local animal care committee. Surgery was performed following i.p. injection of sodium pentobarbital (50 mg/kg). The left jugular vein was catheterized to allow fluid infusion and left femoral artery was cannulated for blood pressure monitoring and blood withdrawal and sampling. Furthermore, tracheotomy was performed and cannulated with a polyethylene-90 tube to facilitate spontaneous breathing. Animals were placed in the supine position on the heating pad to maintain the body core temperature. During experiment, if animals response to a toe pinching, a small bolus of sodium pentobarbital (10-15 mg/kg, i.p.) will be administered.

### Inclusion criteria

Animals under anesthesia were suitable for the experiments if animals had no bleeding and systemic parameters were within normal range, namely (i) mean arterial blood pressure (MAP) above 80 mmHg, (ii) heart rate (HR) above 320 beats/minute, and (iii) systemic hematocrit (Hct) above 45%.

### Systemic parameters and biophysical properties

The MAP was monitored continuously (MP150, Biopac System Inc., Santa Barbara, CA), except during blood exchange. The Hct was determined from centrifuged arterial blood samples taken in heparinized capillary tubes. Viscosity was measured at a shear rate 160 s^-1^(Brookfield Engineering Laboratories, Middleboro, MA). Colloid osmotic pressure (COP) of PE and blood plasma was determined using a membrane colloid osmometer (model 420, Wescor, Logan, UT).

### Cardiac function

The closed chest method was performed to assess cardiac function in this study.[10] The right common carotid artery was exposed allowing a 1.4F pressure-volume conductance catheter (PV catheter; SPR-839, Millar Instruments, Houston, TX) to be inserted. The PV catheter was advanced passing through the aortic valve into the left ventricle. At the baseline and the end of experiment, a bolus of 15% hypertonic saline (10 µl) was intravenously injected to determine the parallel volume (*V*_p_).[11] The pressure and volume signals were instantaneously digitized and acquired (MPVS300, Millar Instruments, Houston, TX, and PowerLab 8/30, ADInstruments, Colorado Springs, CO).

### Moderate isovolemic hemodilution protocol

**Figure 1:**
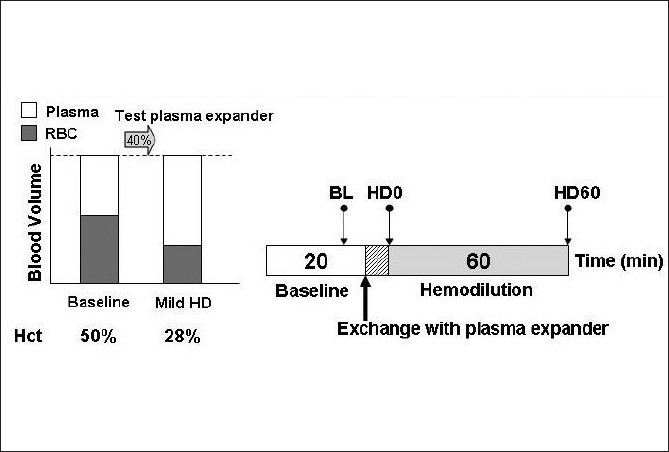
Diagram of the moderate isovolemic hemodilution protocol. Baseline characterization was performed before hemodilution. Blood volume was exchanged for 40% of blood volume with test plasma expanders. Observation period was 1 hr after hemodilution. Hct, hematocrit; BL, baseline; HD, hemodilution

Anesthetized animals were exchanged by 40% of estimated blood volume (BV) with the test solution, lowering systemic Hct by 45%. Total BV was estimated as 7% of body weight. Test solutions were infused into the jugular vein catheter at a rate of 100 µl/min with simultaneous blood withdrawal at the same rate from the femoral artery catheter by using a dual syringe pump (33 syringe pump, Harvard Apparatus, Holliston, MA). Blood samples were collected at the end of experiment for subsequent measurement of viscosity, plasma colloid osmotic pressure, and blood conductance. Animals were monitored for 1 hr after complete hemodilution. Systemic parameters (MAP and Hct) were recorded and analyzed at baseline and 60 min after hemodilution. Figure 1 illustrates the experimental protocol.

### Test solutions

Three solutions were as follows: (1) 6% dextran 2000 kDa (Pharmacosmos, Holbaek, Denmark) in 0.9% sodium chloride (NaCl) mixed with 10% human serum albumin; (2) 6% dextran 70 kDa in 0.9% NaCl (B. Braun Medical, Irvine, CA); and (3) PEG-HSA was prepared by PEGylation conjugated with human serum albumin (HSA; Sigma-Aldrich, St. Louis, MO). The process for PEGylation and conjugation of PEG-HSA was previously described by Meng *et al*.[12] Table 1 lists the physical properties of these test solutions.

**Table 1:** Physical properties of the solutions

Solution	Viscosity (cP)	COP (mmHg)
PEG-HSA	2.2	58
Dx07	3.0	52
Dx2M	6.3	43

Viscosity was measured at shear rate of 160 s^-1^ at 37°C. Values are means.

### Estimation of left ventricular blood volume

**Figure 2:**
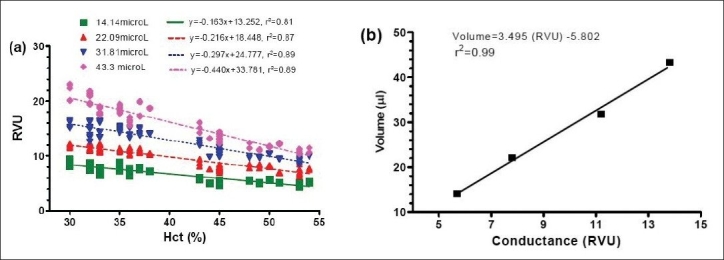
(a) Linear regression of systemic hematocrit and blood conductance (RVU) in each known-volume cylinder. This calibration chart was created using our previous experiments in 20 blood samples for each cuvette. (b) Linear regression of blood calibration from blood conductance (RVU) to actual volume (µl) in each known-volume cylinder

Left ventricular blood volume was measured continuously in conductance units (RVU; relative volume unit) and converted to actual blood volume (µl) at the end of the experiment. The blood calibration was performed using a series of four known-volume cylindrical cuvettes (14.14, 22.09, 31.81, and 43.30 µl). Calibration was established to define the Hct effect in blood conductance, as shown in Figure 2a. Left ventricular blood volume measured by the conductance catheter was determined by using the following equation:

$$V_{\mathrm{lv}}=S\;*\;\mathrm{RVU}\;+\;C\;-V_{p}\;\;\;\;\;\;\;\;\;\;\;\;\ldots \left(1\right)$$

where *V*_lv_is the absolute left ventricular blood volume, RVU is the blood conductance measured by PV catheter, *S* and *C* are the slope and the intercept of linear regression from blood calibration, respectively, as demonstrated in Figure 2b and *V*_p_is the parallel volume caused by the tissues surrounding left ventricle.

### Estimation of total blood volume

The total blood volume at the baseline was approximately about 7% of body weight. In this study, the total blood volume after hemodilution was estimated using the concept of red blood cells (RBCs) conservation. The amount of remaining RBCs after hemodilution was calculated from the difference between the amount of RBCs at baseline (RBC_BL_) and the amount of RBCs withdrawn during blood exchange (RBC_E_). The estimated blood volume (EBV) was calculated from the amount of remaining RBCs and the final Hct (Hct_F_) as:

$$\mathrm{EBV}\;=\;{\mathrm{RBC}}_{\mathrm{BL}}-\;{\mathrm{RBC}}_{E}\;/\;{\mathrm{Hct}}_{F}\;\;\;\;\;\;\;\;\;\;\;\;\ldots \left(2\right)$$

### Data analysis of cardiac function

Cardiac function data were analyzed with the PVAN software (version3.6, Millar Instruments, Houston, TX). Measured cardiac function parameters such as heart rate (HR), end-systolic pressure (*P*_es_), end-diastolic pressure (*P*_ed_), end-systolic volume (*V*_es_), and end-diastolic volume (*V*_ed_) were picked up from the pressure and volume signals. Indices of cardiac function were calculated including maximum rate of pressure change (d*P*/d*t*_max_), minimum rate of pressure change (d*P*/d*t*_min_), relaxation time constant (Tau), cardiac output (CO), stroke work (SW), and stroke volume (SV). The values of studied cardiac function indices were averaged from selected 8-12 cardiac cycles at each time point. Furthermore, systemic vascular resistance (SVR) was calculated from MAP divided by CO to evaluate vascular function.

### Statistical analysis

Results are presented as mean ± standard deviation unless otherwise noted. Data between interested time points in a same group were analyzed using analysis of variance for repeated measurements (ANOVA) and followed by post hoc analyses with the Dunnett’s multiple comparison tests. An unpaired t-test with two-tailed was performed to compare between groups at the time point of interest. All statistics were calculated using GraphPad Prism 4.01 (GraphPad Software, San Diego, CA). Results were considered statistically significant if *P*<0.05.

## Results

A total of 22 animals were entered into the study, they were randomly assigned to the following groups: Dx2M (n=6; 67.8± 4.2 g), Dx70 (n=8; 67.9± 2.0 g) and PEG-HSA (n=8; 66.7± 1.7 g). All animals tolerated the hemodilution protocol for the entire period of experiment. Table 2 compares viscosities of blood and plasma at 60 min after hemodilution. Dx2M reduced blood viscosity to 77% of the baseline, while Dx70 and PEG-HSA decreased blood viscosity to 66% and 60% of the baseline, respectively. Furthermore, Dx2M significantly increased plasma viscosity for 62% and 75% higher than Dx70 and PEG-HSA, respectively (*P*<0.001).

**Table 2:** Rheological properties at 60 min after hemodilution

60 min after hemodilution	n	Hct (%)	Blood viscosity (cP)	Plasma viscosity (cP)	Plasma COP (mmHg)
Blood (Baseline)	3	52 ± 1	4.71 ± 0.62	1.05 ± 0.04	16 ± 2
Dx2M	6	27 ± 2***	3.61 ± 0.24**	2.12 ± 0.10***	18 ± 2
Dx70	8	28 ± 2***	3.09 ± 0.09***	1.27 ± 0.03***†††	16 ± 1
PEG-HSA	8	27 ± 2***	2.84 ± 0.24***†	1.17 ± 0.03**†††	18 ± 2

Viscosity was measured at shear rate of 160 s^-1^ at 37°C. Values are means

** *P*<0.01

*** *P*<0.001 compared with baseline

† P<0.05

††† *P*<0.001 compared with baseline Dx2M

**Figure 3:**
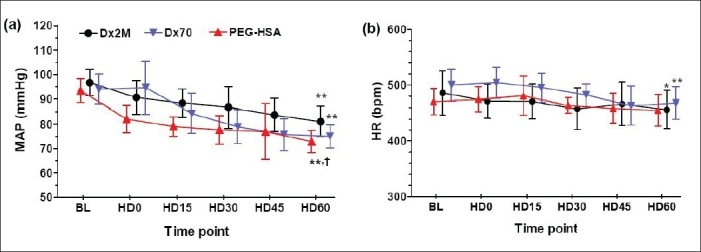
(a) Mean arterial pressure (MAP) and (b) heart rate (HR) measured at baseline (BL), at 0, 15, 30, 45 and 60 minutes after hemodilution (HD0, HD15, HD30, HD45, HD60). Values are presented as means ± SD. *P<0.05; **P<0.01 compared with baseline in a same group. † *P*<0.05 compared with the Dx2M group.

Figure 3a presents MAP at the baseline and during 60 min after hemodilution. Hemodilution drastically decreased MAP by 16%, 21%, and 22% in the Dx2M, Dx70 and PEG-HSA groups at 60 min post-hemodilution relative to baseline, respectively (*P*<0.01). In addition, there was a significant difference in MAP between a group hemodiluted with Dx2M and a group hemodiluted with PEG-HSA at the end of experiment (HD60; *P*<0.05). HR in all groups tentatively decreased over the observation time but only Dx2M and Dx70 caused, at 60 min after hemodilution, a significant drop compared to baseline (*P*<0.05; Figure 3b). Systemic Hct in all groups remarkably reduced by 46-48% after hemodilution (*P*<0.01).

**Figure 4:**
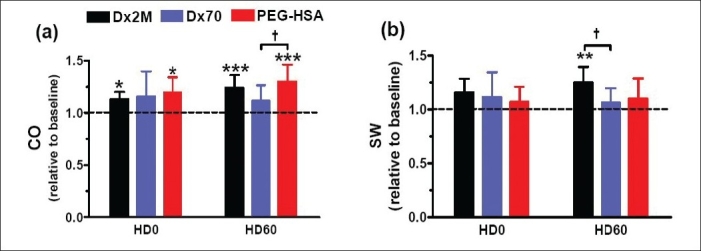
(a) Cardiac output and (b) stroke work at 0 min and 60 min after hemodilution. The baseline mean and standard deviation for cardiac output (CO) is 9.4 ± 2.4 ml/min and stroke work (SW) is 2,134 ± 568 mmHg µl. Broken line represents the values at baseline. Values are presented as means ± SD. * *P*<0.05; **P<0.01; ***P<0.001 compared with baseline. † *P*<0.05 compared between groups

**Figure 5:**
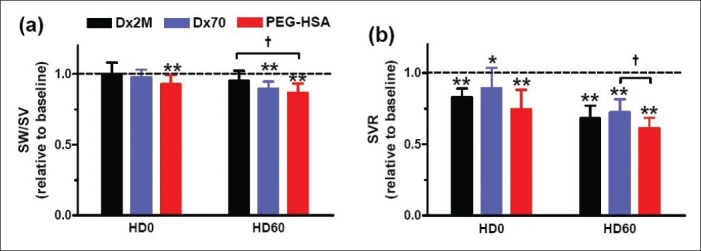
(a) Work done by the heart per stroke volume and (b) systemic vascular resistance at 0 min and 60 min after hemodilution. The baseline mean and standard deviation for work done by the heart per stroke volume (SW/SV) is 110 ± 6 mmHg and systemic vascular resistance (SVR) is 10.8 ± 3.7 mmHg min ml^-1^. Broken line represents the values at baseline. Values are presented as means ± SD. *P<0.05; **P<0.01 compared with baseline. † *P*<0.05 compared between groups

**Figure 6:**
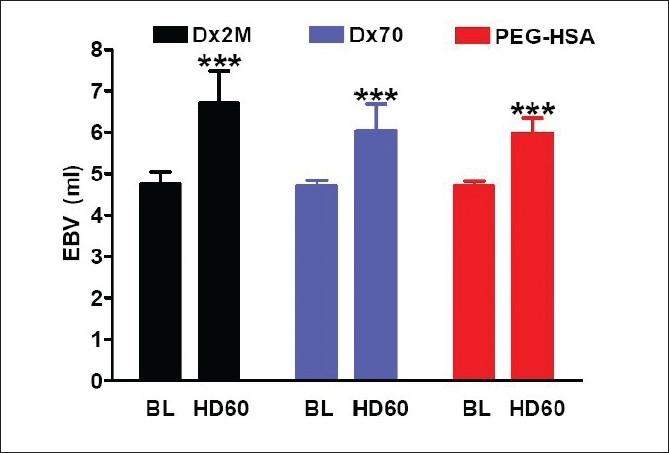
Estimated blood volume (EBV) calculated from RBC volume at 60 min after hemodilution compared with baseline. Values are presented as means ± SD. ***P<0.001 compared with baseline

Table 3 presents left ventricular cardiac indices at the baseline and 60 min after hemodilution. In all groups, hemodilution significantly decreased *P*_es_compared to baseline as well as d*P*/d*t*_min_(*P*<0.05). Only animals exchanged with Dx2M did not have a significant drop in d*P*/d*t*_max_ compared with baseline. Although both Dx2M and PEG-HSA enhanced *V*_ed_, only PEG-HSA significantly enhanced *V*_ed_ at 60 min after hemodilution relative to baseline. The relaxation time constant particularly increased after hemodilution with all test solutions (*P*<0.05). All test solutions also enhanced CO over the baseline value but only Dx2M and PEG-HSA significantly improved, at 60 min after hemodilution, CO compared to baseline (*P*<0.001; Figure 4a). Furthermore, PEG-HSA markedly caused CO by 16% higher than Dx70 (*P*<0.05). By overall, SW elevated after hemodilution with all test solutions and was higher than the baseline over the entire observation period. At 60 min after hemodilution, Dx2M significantly increased SW compared with Dx70 (*P*<0.05; Figure 4b). The work done by the heart per ejected volume (SW/SV) in the Dx2M group slightly dropped when compared with baseline (by 5%), while this ratio significantly decreased relative to baseline by 12% in the Dx70 and PEG-HSA groups (*P*<0.01; Figure 5a). Figure 5b demonstrates that all test solutions potentially lowered SVR compared to baseline (*P*<0.05). At 60 min after hemodilution, PEG-HSA also significantly decreased SVR compared with Dx70 (*P*<0.05). All test solutions significantly increased blood volume after hemodilution in range of 27-41% (*P*<0.001; Figure 6). However, no significant difference in estimated blood volume between groups was found at the end of experiment.

**Table 3:** Cardiac function indices measured and derived from a pressure-volume (PV) measurement

Parameter	Baseline			60 min after hemodilution		
	Dx2M	Dx70	PEG-HSA	Dx2M	Dx70	PEG-HSA
P_es_, mmHg	118.5 ± 6.2	119.0 ± 9.1	113.2 ± 4.1	105.4 ± 9.7**	103.9 ± 8.6**	91.2 ± 7.9**
P_ed_, mmHg	7.6 ± 4.1	6.5 ± 1.8	5.5 ± 1.6	7.6 ± 1.9	8.9 ± 3.1	7.8 ± 3.2*
V_es_, ml	16.5 ± 10.5	13.1 ± 6.9	4.8 ± 2.7	16.1 ± 11.3	8.5 ± 4.9	6.8 ± 3.3
V_ed_, ml	35.6 ± 10.5	28.0 ± 10.2	22.8 ± 4.7	41.5 ± 13.2	26.0 ± 6.9	28.7 ± 7.8*
SV, ml	20.6 ± 3.8	18.1 ± 5.7	20.2 ± 5.5	27.7 ± 8.3**	21.4 ± 8.0*	25.3 ± 5.4**
dP/dt_max_, mmHg/s	12,810 ± 1,194	14,305 ± 1,454	13,391 ± 1,882	12,045 ± 1,460	11,768 ± 1,242**	10,977 ± 1,112**
dP/dt_min_, mmHg/s	−13,981 ± 1,470	−13,021 ± 2,993	−11,614 ± 1,316	−9352 ± 1,770**	−9,821 ± 1,628**	−9,236 ± 1,961*
Tau-Weiss, ms	6.2 ± 1.0	5.8 ± 0.8	6.1 ± 0.5	7.3 ± 1.3**	6.7 ± 1.1*	7.0 ± 0.6*

Values are presented as means ± SD. HR, heart rate; P_es_, end systolic pressure; P_ed_, end diastolic pressure; V_es_, end systolic volume; V_ed_, end diastolic volume; SV, stroke volume; dP/dt_max_, maximum rate of pressure change; dP/dt_min_, minimum rate of pressure change; Tau, relaxation time constant

* *P*<0.05

** *P*<0.01

*** *P*<0.001 compared with baseline

## Discussion

The main finding of this study is that, in moderate isovolemic hemodilution (Hct 28%) and under anesthesia, PEG-HSA (low viscogenic PE) particularly affected on cardiac function as similar as Dx2M (high viscogenic PE) and better than Dx70 (moderate viscogenic PE). Furthermore, PEG-HSA significantly lowered SVR compared with other test PEs. PEG-HSA potentially had beneficial characteristics of PE on the heart performance due to significantly improved CO, SV, and lowered SW/SV ratio, although MAP decreased. Our results also suggest that the beneficial effects of PEG-HSA on cardiac and vascular function were not mainly caused by its viscosity which is obviously unlike Dx2M.

The rheological properties of blood depend on RBC concentration, RBC aggregation, plasma viscosity, and cell deformity. Our results show that an increase of plasma viscosity by viscogenic PEs enhanced blood viscosity toward the normal value, although the Hct decreased. Blood viscosity is a significant factor for endothelial wall shear stress as well as wall shear rate. Cabrales *et al*,[8,13] numerically showed that PEG-HSA led to higher wall shear stress because it provided higher wall shear rate and microvascular blood flow compared to Dx70, although PEG-HSA had lower viscosity than Dx70. Their results supported our finding because, in our study, PEG-HSA significantly enhanced CO relative to Dx70, implying that PEG-HSA improved blood perfusion better than Dx70. Shear stress is a major determinant for the production of vasodilation mediator such as nitric oxide and prostaglandin.[14–16] Therefore, PEG-HSA and Dx2M dilated blood vessel more effectively than Dx70 due to their higher wall shear stress. This conclusion is also supported by Cabrales *et al*.’s study[8] which reported that PEG-HSA increased arteriolar and venular diameters greater than Dx70. Vasodilation in the circulatory system leads to a decrease in SVR and blood pressure. PEG-HSA and Dx2M decreased SVR lower than Dx70 during the observation period, suggesting that PEG-HSA and Dx2M possibly induced more production of vasodilators than Dx70.

The important characteristic of plasma expanders is a volume expansion. The high colloid osmotic pressure of PEs results in shifting of fluids from extravascular space into intravascular space. We calculated the EBV after hemodilution by using the concept that RBCs should be conserved after hemodilution. Our calculations show that blood volume significantly increased relatively to baseline after hemodilution for all PEs. EBV after hemodilution may be under estimation because we did not take account of the ratio of whole-body to large-vessel hematocrit (F-cell ratio) after hemodilution.[17] However, taking account of the F-cell ratio does not change the tendency of EBV.

The load-dependent parameters derived from pressure-volume measurement (dP/d*t*_max_ and d*P*/d*t*_min_) and *P*_es_decreased after hemodilution, suggesting the sign of the depression of cardiac function in systole and diastole. However, this depression is controversial with an increase of CO. At 60 min after hemodilution, Dx70 and PEG-HSA significantly reduced d*P*/d*t*_max_ relative to baseline while Dx2M did not show that. These results indicate that systolic function is more deficient when hemodilution with Dx70 and PEG-HSA. The decrease of *P*_es_ in all groups was accompanied with the reduction of MAP. Furthermore, the decrease of *P*_es_ may be the result of lowering of SVR and after load. Our study also evaluated the fall of left ventricular pressure during isovolumic relaxation phase by the Weiss relaxation time constant (Tau). We found that hemodilution with each PEs increased Tau, indicating a prolong diastolic relaxation and a sign of diastolic function impairment.[18] In the isovolumic relaxation phase, there is ATP usage for calcium uptake by sarcoplasmic reticulum. Thus, hemodilution might delay this process as depicted by an increased Tau.

As the oxygen carrying capacity decreases in hemodilution, the compensatory mechanisms increase the volume flow rate to maintain or sustain the oxygen delivery for tissue oxygenation with limitation. Our findings present that PEG-HSA potentially enhanced and maintained CO higher than the baseline over the observation period as similar as Dx2M, although PEG-HSA had significantly lower viscosity than Dx2M. In our study, HR after hemodilution was relatively lower than the baseline; therefore, the enhancement of CO after hemodilution with PEG-HSA, Dx2M or Dx70 was a result of the increase of SV. Furthermore, PEG-HSA markedly increased an end-diastolic volume (*V*_ed_) while an end-systolic volume (*V*_es_) was not significantly changed at 60 min after hemodilution, resulting in the increase of SV. The increase of *V*_ed_ or preload after hemodilution also caused the heart to work more as shown by the increase of stroke work. Our results further present that an increase of plasma viscosity with Dx2M led to an increase of stroke volume and more work or energy required to pump out blood, unlike PEG-HSA performed. Microvascular studies showed that increase of CO correlated to increase of microvascular blood perfusion and oxygen delivery and extraction.[19–21] In addition, Cabrales and coworkers[8] observed that PEG-HSA increased microvascular oxygen delivery and extraction higher than Dx70 in an extreme hemodilution model. Regarding to their findings, we concluded that, in our study, hemodilution with PEG-HSA and Dx2M provided sufficient oxygen for the heart to perform work and this was better than hemodilution with Dx70.

In our experiments, the blood conductance at the baseline was estimated from the baseline Hct and was used to calculate the volume-related indices such as *V*_es_, *V*_ed_, SV, and SW. This method might give an over or under-estimation of *V*_es_ and *V*_ed_. However, this method did not significantly affect the differential volume-related indices such as SV and SW. Furthermore, the parallel conductance (*V*_p_) determined by a bolus of hypertonic saline injection was not a constant for each animal after hemodilution with PEs. This is another cause that may affect the accuracy of the volume-related cardiac function indices. To minimize the error from the *V*_p_ measurement, a bolus of hypertonic saline was injected when the heart rhythm was regular and the linear regression correlation coefficient of the extrapolation to determine *V*_p_ was higher or equal to 0.8 (r ≥0.8).

We conclude that PEG-HSA has a significant lower viscosity compared to other higher viscogenic PEs, but it efficiently improves cardiac output and preload and lowers pumping work per stroke volume after acute moderate hemodilution. These beneficial effects of PEG-HSA are not the direct results of increase of plasma viscosity, unlike Dx2M and Dx70. Therefore, PEG-HSA possibly has the interaction mechanisms with vasculature different from higher viscogenic PEs, but it causes the similar effects on the cardiovascular system, leading to an interesting issue on its interaction mechanism with vasculature for a further study of this novel plasma expander.
